# NSUN4-mediated m5C RNA methylation protects retinal cells against excitotoxic injury via the SHH signaling pathway

**DOI:** 10.1371/journal.pone.0347414

**Published:** 2026-04-15

**Authors:** Yahong Li, Dian Li, Chao Geng, Ruihua Wei, Yajian Duan

**Affiliations:** 1 Department of Ophthalmology, Shanxi Bethune Hospital, Shanxi Academy of Medical Sciences, Third Hospital of Shanxi Medical University, Tongji Shanxi Hospital, Taiyuan, China; 2 Department of Biochemistry and Molecular Biology, Shanxi Medical University, Taiyuan, China; 3 Washington University School of Medicine, St. Louis, Missouri, United States of America; 4 School of Medicine, Nankai University, Tianjin, China; 5 Tianjin Key Laboratory of Retinal Functions and Diseases, Tianjin Branch of National Clinical Research Center for Ocular Disease, Eye Institute and School of Optometry, Tianjin Medical University Eye Hospital, Tianjin, China; University of Vermont, UNITED STATES OF AMERICA

## Abstract

Glaucoma, a leading global cause of blindness, is characterized by progressive retinal neuronal loss. NOP2/Sun RNA methyltransferase 4 (NSUN4), a writer of 5-methylcytosine (m5C) RNA modifications, has established roles in methylation and mitoribosome assembly, yet its function in retinal cell survival remains unexplored. In this study, integrated methylated RNA immunoprecipitation sequencing (MeRIP-seq) and RNA-seq analysis in an NMDA-induced retinal injury model revealed widespread mRNA hypomethylation enriched in the Sonic Hedgehog (SHH) signaling pathway, accompanied by significant downregulation of *Nsun4*. To investigate the underlying mechanisms, we utilized the R28 retinal cell line, a widely accepted model for studying retinal neuroprotection. In glutamate-stimulated R28 cells, NSUN4 overexpression mitigated excitotoxic injury, attenuating Ca² ⁺ overload, mitochondrial dysfunction, and apoptosis. Mechanistically, NSUN4 enhanced m5C methylation on key SHH pathway transcripts (*Shh, Gli1, and Gli2*). Crucially, the neuroprotective effect of NSUN4 was abolished upon pharmacological inhibition of the SHH pathway using Vismodegib, confirming that pathway activation is essential for NSUN4-mediated protection. Clinically, *NSUN4* levels were significantly reduced in the aqueous humor of patients with primary open-angle glaucoma compared to controls. Together, these findings establish NSUN4 as an m5C-dependent activator of the SHH pathway that protects retinal cells against excitotoxic injury, nominating it as a novel candidate for glaucoma neuroprotection.

## Introduction

Glaucoma is a multifactorial neurodegenerative disease characterized by progressive optic nerve damage and retinal ganglion cell (RGC) death. It is the second leading cause of irreversible blindness worldwide [1,2]. The estimated prevalence of glaucoma in the global population aged 40–80 years is 3.5%, with an expected 111.8 million people affected by glaucoma in 2024 [1]. The pathophysiology of glaucoma is multifactorial and complex [3]. Currently available treatments involving drugs or surgery cannot reverse glaucomatous vision loss [1]. Therefore, novel strategies to reduce or reverse vision loss caused by glaucoma must be explored [4].

One key mechanism implicated in glaucoma progression is glutamate excitotoxicity. As the predominant excitatory neurotransmitter in the central nervous system, glutamate can induce neuronal injury when its signaling is dysregulated. Overstimulation of N-methyl-d-aspartate (NMDA) receptors leads to disrupted ionic homeostasis and sustained calcium influx [2]. This in turn triggers a cascade of deleterious events including mitochondrial dysfunction, oxidative stress, and ultimately RGC death—a process recognized as a central pathway in glaucoma pathogenesis [4–6]. Accordingly, NMDA receptor activation has been widely used in rodent models to mimic RGC excitotoxicity and screen potential neuroprotective agents [6,7], positioning it as a promising therapeutic target for glaucoma and other neurodegenerative conditions [4].

The Sonic Hedgehog (SHH) signaling pathway mediates its biological effects by inducing the transcription of downstream GLI family proteins (GLI1, GLI2, and GLI3) and other SHH-targeted genes [8]. Beyond its established role in neural development, the SHH pathway is crucial for maintaining cellular homeostasis and has been increasingly implicated in neuroprotection. Studies indicate that SHH signaling supports neural tissue through multiple mechanisms, including the promotion of neurogenesis, mitigation of oxidative stress, prevention of mitochondrial dysfunction, suppression of inflammation, and regulation of apoptosis [9–12]. Notably, in models of chronic hypertension, SHH activation has been shown to exert protective effects on damaged retinal ganglion cells (RGCs) via GLI1 activation, highlighting its relevance in glaucoma pathology [13]. Further supporting its therapeutic potential, recent evidence demonstrates that SHH signaling can modulate the behavior of primary human trabecular meshwork (TM) cells in a GLI1-dependent manner [14], suggesting its utility as a target for alleviating cellular injury in glaucoma.

Alongside signaling pathways, post-transcriptional RNA modifications such as 5-methylcytosine (m5C) have emerged as important regulators of gene expression in neurological and mitochondrial contexts [15–17]. Among m5C writers, NSUN4, known as a mitochondrial methyltransferase required for ribosomal assembly and translation, stands out for its established role in maintaining mitochondrial function [18]. Mitochondrial integrity is well established as vital for the health and survival of retinal neurons. Notably, to our knowledge, no prior studies have directly investigated the role of m5C modifications in glaucoma or the functional relationship between m5C methyltransferases such as NSUN4 and the SHH signaling pathway in the retina. Given that mitochondrial dysfunction is a recognized hallmark of glaucoma, we hypothesize that NSUN4 may play a crucial role in the pathogenesis of this disease, potentially linking RNA epitranscriptomic regulation to retinal neurodegeneration.

To validate our hypothesis, an *in vivo* NMDA-induced excitotoxicity model and an *in vitro* glutamate-induced R28 cell line were used in this study. Using MeRIP-seq combined with RNA-seq, we profiled alterations in m5C methylation and transcriptomic landscapes in the retina under excitotoxic conditions. Our analysis revealed a predominant pattern of mRNA hypomethylation in the NMDA-treated group compared to controls. We further demonstrated that NSUN4 plays a critical role in preventing Ca^2+^ overload, mitochondrial dysfunction and cellular apoptosis in retinal neuronal-like cells in a m5C-dependent manner. Mechanistically, NSUN4 enhanced both the m5C methylation and transcript levels of *Shh*, *Gli1*, and *Gli2* in the SHH signaling pathway. Crucially, the neuroprotective effect of NSUN4 was abolished by the SHH pathway inhibitor Vismodegib, confirming that NSUN4-mediated protection is functionally dependent on SHH signaling activation. Furthermore, clinical analysis revealed a significant downregulation of NSUN4 in the aqueous humor of patients with primary open-angle glaucoma (POAG), underscoring its clinical relevance. Collectively, our findings indicate that NSUN4 protects retinal neurons against excitotoxic injury by regulating the SHH signaling pathway via m5C-dependent mechanisms, thereby providing novel insights into the role of RNA methylation in glaucoma.

## Materials and methods

### Animals and NMDA-induced retinopathy rat model

Forty specific pathogen-free Sprague‒Dawley (SD) rats at 7−8 weeks of age were purchased from Beijing Vital River Laboratory Animal Technology Co., Ltd. (Beijing, China). The rats were raised under a 12/12 h light/dark cycle at 22−25°C and a relative humidity of 38−50% and were provided ad libitum access to food and water. All animal experiments were performed in compliance with the ARVO Statement for the Use of Animals in Ophthalmic and Vision Research. The animal experiment procedures were approved by the Institutional Animal Care and Use Committee of Tianjin Medical University (Permit Number: SYXK 2018-0004), and were performed in accordance with the Guide for the Care and Use of Laboratory Animals of the National Institutes of Health. All authors complied with the ARRIVE guidelines. The sample size for animals was calculated using the resource equation method [19].

All rats were randomly divided into two groups (n = 20 each): normal control and NMDA (Sigma, Sigma‒Aldrich, St. Louis, MO, USA) (50 mM). All the rats were anesthetized by intraperitoneal injection of pentobarbital sodium, and all efforts were made to minimize suffering. Oxybuprocaine hydrochloride (Santen Pharmaceuticals, Tokyo, Japan) was used for ocular surface anesthesia. A microsyringe (Hamilton, Reno, NV, USA) with a 33 G needle was inserted into the vitreous cavity, and 3 μL of saline (Otsuka Pharmaceutical Co., Ltd., China) or NMDA solution was injected into the right eye. All surgical procedures were performed under a stereomicroscope. Levofloxacin eye drops (Santen Pharmaceuticals, Tokyo, Japan) were used to prevent infection after injection. After 7 days of treatment, the treated rats were euthanized by an anesthetic overdose, and retinas of the rats were collected for follow-up research. The euthanasia of experimental animals in this research was performed in accordance with internationally recognized guidelines on animal welfare. Once the animals were deeply anesthetized, cervical dislocation was swiftly performed by a trained technician, ensuring a rapid and painless death.

### Sample preparation for sequencing analyses

For both MeRIP-seq and RNA-seq analyses, retinal tissues were processed using an identical pooled-sample strategy to ensure direct comparability between the two omics datasets. For each experimental group (Control and NMDA-treated), retinal tissues from three rats were pooled and thoroughly homogenized to create a single, biologically representative composite sample. This composite sample was then divided into two equal technical aliquots. One aliquot was dedicated to MeRIP-seq library construction for epitranscriptomic profiling, and the other aliquot was processed in parallel for RNA-seq library construction for transcriptomic profiling. This design ensured that both datasets originated from an identical biological source, facilitating a direct and internally controlled comparison of m⁵C modification and gene expression levels.

### Methylated RNA immunoprecipitation sequencing (MeRIP-seq)

MeRIP-seq and subsequent data analyses were mainly supported by CloudSeq Biotechnologies, Inc. (Shanghai, China). Total RNA was extracted from the dedicated MeRIP-seq aliquot (as described above). The quality and quantity of total RNA were assessed by using a NanoDrop ND-1000 (Thermo Fisher Scientific, Waltham, MA, USA). Then, m5C RNA immunoprecipitation was performed with the GenSeqTM m5C RNA IP Kit (GenSeq Inc., China) following the manufacturer’s instructions. Both the input samples without immunoprecipitation and the m5C IP samples were subjected to RNA-seq library generation with the NEBNext® Ultra II Directional RNA Library Prep Kit (New England Biolabs, Inc., USA). Library quality was evaluated with a BioAnalyzer 2100 system (Agilent Technologies, Inc., USA). An Illumina HiSeq 4000 sequencer was used to harvest paired-end reads, and Q30 was used for control quality. Then, the clean reads of all the libraries were aligned to the reference genome (UCSC RN5) by HISAT2 software (v2.0.4). Methylated sites on RNAs (peaks) and differentially methylated sites were separately determined by MACS software and diffReps. Peaks overlapping with exons of mRNA were further extracted by customized scripts. The edgeR software (v4.0.16) was used to analyze fold-expression changes and false discovery rate (FDR) [20]. The FDR was controlled by applying the Benjamini-Hochberg method. The differentially methylated sites (|fold change| ≥ 2 and *P* value  <  0.00001) were subjected to Gene Ontology (GO) and Kyoto Encyclopedia of Genes and Genomes (KEGG) pathway enrichment analyses.

### Bulk RNA sequencing

Total RNA was extracted from the dedicated RNA-seq aliquot (as described in the Sample Preparation section). Subsequently, the rRNAs were removed from the total RNA by using Ribo-Zero rRNA Removal Kits (Illumina, San Diego, CA, USA). RNA libraries were constructed by using rRNA-depleted RNAs with a TruSeq Stranded Total RNA Library Prep Kit (Illumina, San Diego, CA, USA). Libraries were controlled for quality and quantified using the BioAnalyzer 2100 system (Agilent Technologies, Inc., USA). Then, an Illumina HiSeq sequencer was used to comprehensively detect mRNA in the samples. Paired-end reads were harvested from the Illumina HiSeq 4000 sequencer and checked using a quality score of Q30. After 3’ adaptor trimming and low-quality read removal by Cutadapt software (v1.9.1), the high-quality trimmed reads (clean reads) were aligned to the rat reference genome (UCSC RN5) with HISAT2 software (v2.0.4). Then, the Ensembl GTF gene annotation file was used with Cufflinks software to calculate FPKM (Fragments Per Kilobase of transcript per Million mapped reads) values as the expression profiles for each sample. To provide a quantitative estimate of expression changes, read counts were also generated and processed using the edgeR software (v4.0.16) [20]. As the experimental design involved only one pooled library per condition, there were no biological replicates to support statistical inference of differential expression (e.g., calculation of p-values or FDR). Therefore, we adopted an effect size-based approach for initial candidate screening. The fold change in expression between conditions was calculated directly from the FPKM values. mRNAs with a |fold change| ≥ 2 and an average FPKM ≥ 0.1 were selected as putatively differentially expressed for further analysis. Gene Ontology (GO) and pathway enrichment analyses were performed on this gene set. These RNA-seq results are presented as exploratory findings to guide hypothesis generation and subsequent validation, not as statistically confirmed differential expression.

### R28 cell culture and glutamate-induced R28 cell line model

The R28 cells, an immortalized adherent retinal precursor cell line, were generously provided from the Eye Center of Xiangya Hospital, Central South University (Changsha, China). The cells were cultured in low-glucose Dulbecco’s modified Eagle’s medium (DMEM) (Gibco, Waltham, MA, USA) supplemented with 10% fetal bovine serum (Gibco, Waltham, MA, USA) and 1% penicillin‒streptomycin (Gibco, Waltham, MA, USA). The cultured cells were maintained at 37°C with 5% CO_2_ in a humidified incubator. According to the literature [21,22], the cells in the glutamate group were treated with 10 mM L-glutamate (ab120049, Abcam, Cambridge, UK) and incubated for 24 h.

### Quantitative real time polymerase chain reaction (qRT-PCR)

Total RNA was isolated from R28 cells using TRIpure Total RNA Extraction Reagent (ELK Biotechnology, Denver, CO, USA). Subsequently, cDNA was synthesized utilizing EntiLink™ 1st Strand cDNA Synthesis Super Mix (ELK Biotechnology, Denver, CO, USA). Quantitative real-time polymerase chain reaction (qRT‒PCR) was performed using EnTurbo™ SYBR Green PCR SuperMix (ELK Biotechnology, Denver, CO, USA) with three biological replicates. The specific primers used were purchased from Wuhan GeneCreate Co., Ltd. (Wuhan, China). The primer information is provided in Table 1. Relative mRNA levels were normalized to those of *β-Actin* and were calculated using the 2^−ΔΔCT^ method.

**Table 1:** Primers used for quantitative real-time-PCR.

Genes	Species		Sequences (5`-3`)
*Nsun4*	Rat	Forward	CTCTCAGAGCAGAAGTATGGTGC
		Reverse	GATAGAGTTGGAGAGAGGCCACT
*Shh*	Rat	Forward	CGATATGAAGGGAAGATCACAAG
		Reverse	TGCTCGACCCTCATAGTGTAGAG
*Gli1*	Rat	Forward	GACTTGAGCATTATGGACAAGTG
		Reverse	CTGGATTCTAACGAGAGACAGTCAT
*Gli2*	Rat	Forward	GTTCAGCCTTTGGACATACACC
		Reverse	TCGCTGTTCTGCTTATTCTGGT
*Ptch1*	Rat	Forward	TACATGTATAACAGGCAATGGAAGT
		Reverse	GCAGTCCAAAGGTGTAATGATTAAG
*Smo*	Rat	Forward	CAGATGGCACCATGAGATTTG
		Reverse	GAGCAGGTGGAAATAGGATGTC
*β-Actin*	Rat	Forward	CGTTGACATCCGTAAAGACCTC
		Reverse	TAGGAGCCAGGGCAGTAATCT
*NSUN4*	Human	Forward	CCTTCATGAGGAGGAGAACAAC
		Reverse	GTGAGCAGGTAGAATAGACAACATG
*β-ACTIN*	Human	Forward	GTCCACCGCAAATGCTTCTA
		Reverse	TGCTGTCACCTTCACCGTTC

Note: *Nsun4*: NOP2/ Sun RNA methytransferase 4; *Shh*: Sonic Hedgehog; *Gli1*: GLI family zinc finger 1; *Ptch1*: Patched 1; *Smo*: Smoothened.

### Construction of the Nsun4 ORF plasmid

The full-length open reading frame (ORF) plasmid of the rat *Nsun4* gene (NM_001106678.1) vector was purchased from ELK Biotechnology Co., Ltd. (Wuhan, China). The *Nsun4* plasmid DNA was purified from the bacterial culture using an EndoFree Plasmid Miniprep Kit (EP004, ELK Biotechnology, Wuhan, China). Then, the plasmid was digested through the restriction endonuclease sites KpnI and XhoI and inserted into the pcDNA 3.1 vector by T4 DNA ligase to construct overexpression plasmids. For DNA assembly, positive colonies were cultured in Luria–Bertani medium overnight. The plasmid DNA was further amplified and extracted by PCR using Entilink^TM^ PCR Master Mix (EQ001, ELK Biotechnology, Wuhan, China) and Sanger sequencing in GeneCreate (Wuhan, China). S1 Table shows the cloning primers used.

### Transfection of R28 cells

R28 cells were cultured in a 6-well plate at a density of 5 × 10^5^cells/well with antibiotic-free culture medium to 60–70% confluency. The cells were divided into 3 groups: control, empty vector, and NSUN4. For transfection, a transfection mixture containing *Nsun4* plasmids at a concentration of 2 μg/cell was prepared using Opti-MEM™ I Reduced Serum Medium (Gibco, Waltham, MA, USA) and Lipofectamine 2000 (Thermo Fisher Scientific, Waltham, MA, USA), according to the manufacturer's instructions. This mixture was applied to the cells in the NSUN4 group. In parallel, an empty vector was transfected into R28 cells as a control, following the same procedure. At 6 hours post-transfection, the medium was replaced with complete DMEM. Cells were harvested via trypsin digestion 48 hours post-transfection. Transfection efficiency was verified using qRT‐PCR. All experiments, including those involving empty vector transfection controls, were performed with n = 3 independent biological replicates to ensure reproducibility and statistical reliability.

### Cell viability

A cell counting kit-8 (CCK8) assay (Beyotime, Shanghai, China) was performed to determine cell viability. R28 cells were seeded into 96-well plates at a density of 1 × 10^4^ cells/well and then treated with glutamate and/or NSUN4. For experiments involving the Hedgehog pathway inhibitor Vismodegib, the specific treatment groups were: Control, Glu, Glu + NSUN4, and Glu + NSUN4 + Vismodegib. Specifically, cells in the Glu + NSUN4 + Vismodegib group were pretreated with 1 μM Vismodegib for 1 hour prior to the application of glutamate and the induction of NSUN4 overexpression. The other groups (Control, Glu, and Glu + NSUN4) did not undergo this pretreatment step. Each experimental condition was assayed in six technical replicates. Subsequently, 10 μL of a solution containing 10% CCK-8 reagent was added to each well, and the plates were incubated for an additional 3 h in the dark at 37°C. The absorbance at 450 nm was measured using a DR-200Bs microplate reader (Diatek, Wuhan, China) to determine the optical density (OD). The cell viability was calculated using the following formula: (OD_treated_ - OD_blank_) / (OD_control_ - OD_blank_) × 100%.

### Calcium content

The calcium content of the R28 cells in the three groups was measured as previously described [23]. Cells seeded in 6-well plates were fully lysed, with each group comprising three replicate wells. After centrifugation at 14,000 × g for 5 min at 4°C, the supernatant was removed and then analyzed using the o-cresolphthalein complexone method to detect calcium deposition at 575 nm with a microplate reader (USCNK, Hubei, China) in accordance with the instructions of the calcium colorimetric assay kit (Beyotime, Shanghai, China).

### Apoptosis assay with Annexin V-FITC/PI

Flow cytometry was utilized to detect cell apoptosis using Annexin V-FITC/propidium iodide (PI) staining (Sangon Biotech, Tianjin, China). The R28 cells were suspended in binding buffer and then labeled with 5 μl of Annexin V‐FITC along with 5 μl of PI at room temperature for 15 min in the dark. The experiment was independently repeated three times (n = 3). The percentage of apoptotic cells was quantified and analyzed using a flow cytometer (Beckman Coulter, CytoFLEX, USA).

### Analysis of the mitochondrial membrane potential

The mitochondrial membrane potential (MMP) was analyzed using a MMP assay kit with JC-1 (Beyotime, Shanghai, China). 5 × 10^5^ R28 cells were seeded in a 6-well plate, washed with phosphate-buffered saline (PBS), incubated with JC-1 solution at 37°C in the dark for 30 min, and washed with PBS. Then, 4’,6-diamidino-2-phenylindole (DAPI) was used to stain the nucleus, which was incubated at room temperature in the dark for 30 min and washed with PBS. The cells were observed using a fluorescence microscope (Nikon, Eclipse Ci-L, Japan). The experiment was independently repeated three times (n = 3). The relative ratio of red/green fluorescence was used to evaluate mitochondrial damage.

### Dot blot assay

Total mRNA from R28 cells was purified with TRIpure Total RNA Extraction Reagent (EP013, ELK Biotechnology, Denver, CO, USA). The experiment was independently repeated three times (n = 3). Two microliters of mRNA solution were loaded on a polyvinylidene fluoride (PVDF) membrane (Millipore, Massachusetts, USA) and irradiated with UV for 10 min for sample fixation. Then, the membrane was washed in wash buffer (1x TBS and 0.02% Tween-20) for 5 min. The m5C antibody (Ab214727, Abcam, Cambridge, UK) was diluted 1:1000 in 5% nonfat milk in wash buffer. After overnight incubation at 4°C, the membrane was washed three times gently in wash buffer for 5 min. The secondary antibody (HRP-goat anti-rabbit; AS1107, ASPEN, Wuhan, China) was diluted 1:10,000 in wash buffer and incubated for 1 h at room temperature. Then, an enhanced chemiluminescence (ECL) mixed solution (AS1059, ASPEN, Wuhan, China) was added to the sample surface of the membrane, which was exposed in a dark room. The membrane was stained with 0.02% methylene blue (M8030, Solarbio, Beijing, China).

### MeRIP-qPCR

A methylated RNA immunoprecipitation (MeRIP) assay was performed using an EZ-Magna RIP Kit (17–701, Millipore, Germany) following the manufacturer’s instructions, with three independent biological replicates (n = 3). R28 cells were washed with cold PBS and centrifuged for 5 min. Then, R28 cells were lysed using RIP lysis buffer supplemented with protease inhibitor cocktail and RNase inhibitor, followed by incubation on ice for 5 min. The supernatants were collected and incubated with 5 μg of m5C antibody (ab214727, Abcam, Cambridge, UK) or negative control normal anti-rabbit IgG (ab172730, Abcam, Cambridge, UK) to capture the RNAs used for quantitative polymerase chain reaction (qPCR). Each magnetic bead-antibody complex was added to RNA-binding protein-RNA complex immunoprecipitation buffer (860 µl of RIP wash buffer, 35 µl of 0.5 M EDTA, and 5 µl of RNase inhibitor for each reaction). Then, the RNA‒protein IP complexes were rotated for 3 h overnight at 4 °C and washed 6 times with cold RIP Wash Buffer. All tubes were incubated with proteinase K buffer to digest the proteins. Finally, targeted RNAs were extracted from the immunoprecipitated complex, and the relative gene expression of *Shh*, *Gli1* and *Gli2* was measured by qPCR. The primer information used is provided in Table 2. The relative enrichment was normalized to the input = 2^Ct [IP] – Ct [input]^.

**Table 2:** Primer used for MeRIP-qPCR.

Genes		Sequences (5`-3`)
*Shh*	Forward	CGATATGAAGGGAAGATCACAAG
	Reverse	TGCTCGACCCTCATAGTGTAGAG
*Gli1*	Forward	GACTTGAGCATTATGGACAAGTG
	Reverse	CTGGATTCTAACGAGAGACAGTCAT
*Gli2*	Forward	GTTCAGCCTTTGGACATACACC
	Reverse	TCGCTGTTCTGCTTATTCTGGT

Note: *Shh*: Sonic Hedgehog; *Gli1*: GLI family zinc finger 1.

### Aqueous humor collection from patients

Aqueous humor (AH) samples were collected from patients with cataract or glaucoma (n = 3). For the clinical component of this research, the observational study involving human participants was conducted in accordance with the principles of the Declaration of Helsinki following approval from the Institutional Review Board of Shanxi Bethune Hospital (Approval No. LYLL-2025–005/PJ93). Written informed consent was obtained from all participants prior to sample collection. All patients fasted for at least 8 hours overnight as a standard preoperative requirement. At the beginning of ocular surgery, approximately 100–200 μL of AH was aspirated via limbal paracentesis. A 30-gauge needle connected to a small syringe was introduced into the peripheral anterior chamber at the corneoscleral limbus. The needle was advanced in a plane parallel to the lens to avoid injury or hemorrhage, and AH was carefully withdrawn from the mid-anterior chamber. The samples were then transferred into 1.5 mL Eppendorf tubes and immediately stored at −80°C until further analysis.

### Statistical analysis

Data analysis was performed using GraphPad Prism 9.0 software (GraphPad Software, Inc., Boston, MA, USA). The results are presented as the mean  ±  standard deviation (SD). The assumptions of normality and homogeneity of variances for parametric tests were assessed prior to analysis. Normality was tested using the Shapiro-Wilk test, and homogeneity of variances was tested using Levene’s test. Based on these assessments, parametric tests were applied as follows: Unpaired Student’s *t* tests were used for two group comparisons. For comparisons among more than two groups, one-way analysis of variance (ANOVA) was performed, followed by Tukey’s post hoc test for multiple comparisons. For categorical variables (e.g., gender distribution), Fisher’s exact test was employed. *P* value of less than 0.05 was considered statistically significant. To prevent confirmation bias, the investigators were blinded during the outcome assessment and data analysis phases.

## Results

### MeRIP-seq analysis of the NMDA-treated retinal excitotoxic model

In our previous study, an NMDA-induced excitotoxicity model was established in the retina of Sprague Dawley (SD) rats [24]. More specifically, the arrangement of the cells in the ganglion cell layer (GCL) was disrupted 7 days after the intravitreal injection of 50 mM NMDA, resulting in nuclear pyknosis. Moreover, the number of RGCs in the retina of the NMDA-treated group was significantly lower than that in the control group. Furthermore, NMDA injection caused retinal dysfunction via excitotoxicity [24]. Then, we used MeRIP-seq and RNA-seq to determine the role of m5C mRNA modification in the NMDA group.

**Fig 1:**
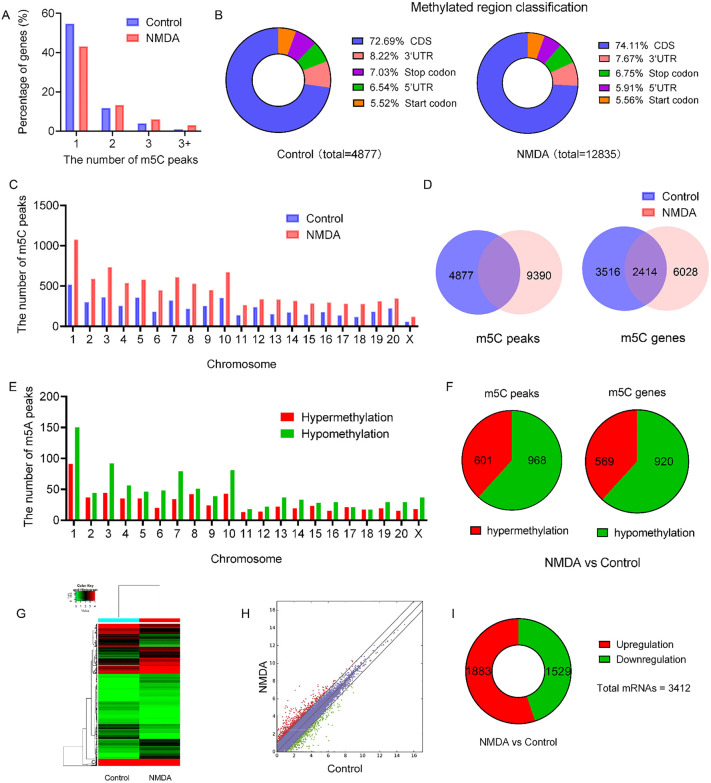
MeRIP-seq and RNA-seq analysis of the two groups. **A.** Number of m5C peaks per gene. More than 40% of the mRNAs contained only one m5C peak. **B.** Pie charts showing the distribution of m5C peaks in different gene contexts. Each transcript is divided into five parts: 5 untranslated regions, coding DNA sequences, 3 untranslated regions, initiation codons, and termination codons. **C.** Distribution of all m5C sites on chromosomes in the control and NMDA groups. **D.** Number of m5C peaks in all mRNAs and mRNAs in the control group and NMDA group. **E.** Distribution of significantly differentially methylated m5C sites on chromosomes in the NMDA group compared to those in the control group (|fold change| ≥ 2 and *P* value  <  0.00001). **F.** Number of significantly differentially methylated m5C sites in the NMDA group. **G.** Color histograms of differentially expressed genes (|fold change| ≥ 2 and FPKM ≥ 0.1) between the control and NMDA groups. The color scale represents gene expression levels. **H.** Scatter plots of the DEGs between the two groups. The red dots indicate upregulation, while the green dots indicate downregulation. The threshold for multiple changes is 2.0. **I.** Pie chart showing the DEGs following NMDA treatment. DEG: differentially expressed genes. Retinal tissues from three rats were pooled to form a single composite sample for sequencing analysis.

A total of 54.75% and 43.21% of the genes in the control group and NMDA group, respectively, exhibited one m5C methylation peak. A total of 11.89% and 13.37% of the genes in each group had two methylation peaks. Fewer genes had three or more methylation peaks (Fig 1A). The identified m5C peaks were not uniformly distributed across the entire transcriptome. The three most enriched genomic regions for both groups were coding sequences (CDSs), 3’ untranslated regions (3’ UTRs), and stop codons (Fig 1B). The number of m5C methylation peaks in the CDS region was the highest, representing more than two-thirds of the methylated mRNAs. Peaks in the stop codon and 3’ UTR regions were found in 8.22% and 7.67% of all m5C-methylated mRNAs, respectively (Fig 1B). The altered m5C peaks were further transcribed from all chromosomes, especially chromosome 1 (chr1) (Fig 1C). Specifically, 4877 and 9390 m5C peaks were identified in the control and NMDA groups, respectively. Furthermore, 3516 and 6028 genes were separately methylated in the two groups. Next, 2414 genes were identified as overlapping genes between the two groups (Fig 1D).

Compared with those in the control group, differentially methylated m5C peaks were identified on all chromosomes (especially chr1) in the NMDA group (Fig 1E). Compared with the control group, the NMDA group exhibited 601 significantly hypermethylated and 986 significantly hypomethylated m5C peaks, defined by an absolute fold change ≥ 2 and a *P* value  <  0.00001. Compared with those in the control group, there was an increase in the methylation (hypermethylation) of 569 genes and a decrease (hypo-methylation) in 920 genes after exposure to NMDA (Fig 1F).

### RNA-seq analysis of the NMDA-treated RGC damage model

To further investigate the mechanisms of retinal damage, bulk RNA-seq was performed on isolated retinas from the NMDA-treated rats and the control rats. Cluster and scatter plots visualizing the sequencing analysis reveal distinct mRNA expression profiles (|fold change| ≥ 2 and FPKM ≥ 0.1) between the control and NMDA-treated groups (Fig 1G and 1H). Among these, 3,412 genes met the criteria for differential expression, with 1,883 upregulated and 1,529 downregulated in the NMDA group compared to the control (Fig 1I). Three types of enzymes implicated in RNA m5C methylation were analyzed: methyltransferases (NSUNs), demethylases (ten-eleven translocation (TETs)), and m5C recognition ’reader’ proteins (YTH domain-containing family 2 (YTHDF2), Aly/REF export factor (ALYREF), and Y-box binding protein 1 (YBX1)) [25]. As shown in Table 3, the expression of the writers *Nsun6* and *Nsun7*, the erasers *Tet3* and *Tet2*, and the readers *Ybx1* and *Ythdf2* was upregulated, while the expression of the writers *Nsun4* and *Nsun5* and *Nsun2*, the eraser *Tet1*, and the reader *Alyref* was downregulated in the NMDA group compared to the control group. Because the MeRIP results showed that there were more hypermethylated mRNAs than hypomethylated mRNAs following NMDA exposure (Fig 1G), the m5C regulators resulting in hypomethylation were further analyzed. Corresponding m5C modifications were observed for the writers *Nsun4*, *Nsun5*, *Tet1*, and *Nsun2* and the erasers *Tet3* and *Tet2* (Table 3). The two different locations of *Nsun4* on chr 5 were shown, and the transcript abundance of *Nsun4* in the NMDA-treated retinas was 3.18-fold less than that in the control group (Table 3). Given that NSUN4 exhibited the most dramatic changes in expression in response to NMDA, NSUN4 was selected as a susceptible target for the NMDA-induced excitotoxicity model.

**Table 3:** The mRNA expression level of m5C regulators in the NMDA group compared to the control group in RNA-seq.

Gene	Regulation	Fold change	log_2_(fold change)	Regulation	GeneID	Chromosome
*Nsun4*	writer	−3.178641	−1.66841	down	298426	5
*Nsun5*	writer	−1.459383	−0.545359	down	288595	12
*Tet1*	eraser	−1.417804	−0.503658	down	309902	20
*Nsun2*	writer	−1.242993	−0.313818	down	361191	1
*Nsun4*	writer	−1.074581	−0.103774	down	298426	5
*Alyref*	reader	−1.008412	−0.012086	down	690585	10
*Tet3*	eraser	1.005111	0.007356	up	680576	4
*Tet2*	eraser	1.015189	0.021749	up	310859	2
*Ybx1*	reader	1.044638	0.063003	up	500538	5
*Ythdf2*	reader	1.059622	0.08355	up	313053	5
*Nsun6*	writer	1.310752	0.390395	up	307148	17
*Nsun7*	writer	1.557653	0.639374	up	305339	14

Note: *Nsun4*: NOP2/Sun RNA methyltransferase family member 4; *Tet1*: Tet methylcytosine dioxygenase 1; *Alyref*: Aly/REF export factor; *Ybx1*: Y box binding protein 1; *Ythdf2*: YTH N(6)-methyladenosine RNA binding protein 2.

### Integrated analysis reveals concurrent m5C hypomethylation and transcriptional downregulation of the Hedgehog signaling pathway under NMDA-induced excitotoxicity

**Fig 2:**
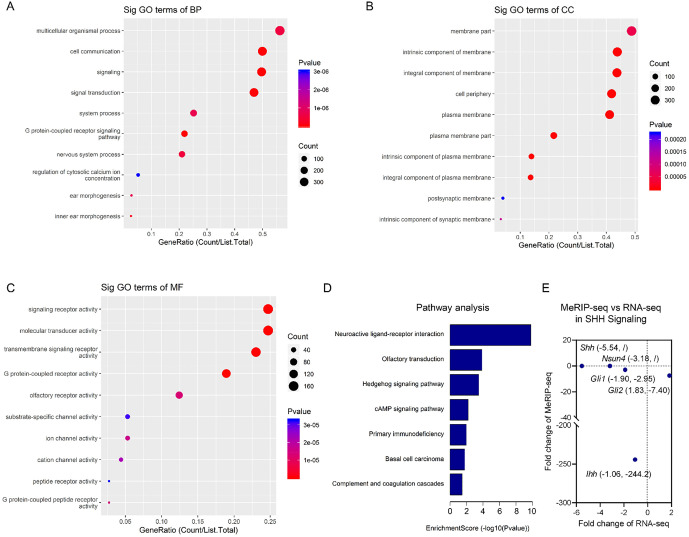
MeRIP-seq analysis of retinas after NMDA treatment. A–C. GO analysis of the hypomethylated mRNAs in the NMDA group compared with those in the control group. **D.** KEGG analysis of hypomethylated mRNAs in the NMDA group compared with the control group. For sequencing analysis, retinal tissues from three rats were pooled together as one sample.

To investigate how dynamic changes in m5C modification regulates gene expression in response to excitotoxicity, we first characterized the functional landscape of genes exhibiting significant m5C hypomethylation in the NMDA-treated group. GO analysis revealed that these hypomethylated genes were significantly enriched in biological processes related to cell communication, signal transduction, and regulation of cytosolic calcium ion concentration (Fig 2A–2C). Notably, the m5C writer Nsun4 was enriched in mitochondrial compartments and identified as a positive regulator of mitochondrial translation (S2 Table). KEGG pathway analysis further identified the Hedgehog (HH) signaling pathway as a top enriched category among hypomethylated genes (Fig 2D), with key components such as *Indian Hedgehog* (*ihh*), G*li1*, and G*li2* prominently represented (Table 4). Given this coordinated epigenetic downregulation of the HH pathway, we next asked whether it was associated with corresponding changes at the transcriptional level. Consistent with this hypothesis, RNA-seq analysis of the same samples showed that key HH pathway genes (*Shh*, *Ihh*, and *Gli1*) were indeed significantly downregulated in the NMDA group (Table 5).

**Table 4:** The m5C information of *Ihh*, *Gli1* and *Gli2* mRNAs in Hedgehog signaling pathway in the NMDA group compared to the control group in MeRIP-seq.

Genes	Foldchange	FDR	Regulation	Gene ID	Chromosome
*Ihh*	−244.2	1.40E-06	down	84399	9
*Gli1*	−2.949446	0.000126	down	140589	7
*Gli2*	−7.398165	6.11E-06	down	304729	13
*Lrp2*	−3.743295	4.36E-06	down	29216	3
*Wnt3*	−6.480799	8.45E-06	down	24882	10
*Wnt3a*	−8.878613	5.5E-12	down	303181	10
*Wnt5a*	−9.92145	1.36E-05	down	64566	16
*Wnt6*	−272.3	2.8E-07	down	316526	9

Note: *Ihh*: Indian Hedgehog; *Gli1*: GLI family zinc finger 1; *Lrp2:* LDL receptor related protein 2; *Wnt3*: wingless-type MMTV integration site family, member 3. FDR: false discovery rate.

**Table 5:** The mRNAs expression of HH signaling pathway in the NMDA group compared to the control group in RNA-seq.

Gene	Fold change	log_2_(fold change)	FDR	Regulation	GeneID	chromosome
*Shh*	−5.541206	−2.4702	0.997307	down	29499	4
*Ihh*	−1.061055	−0.085499	1	down	84399	9
*Gli1*	−1.901752	−0.927329	1	down	140589	7
*Gli2*	1.828649	0.870778	0.997307	up	304729	13

Note: *Shh*: Sonic Hedgehog; *Ihh*: Indian Hedgehog; *Gli1*: GLI family zinc finger 1.

To establish a functional link between m⁵C epitranscriptomic alterations and gene regulation, we performed a conjoint analysis of MeRIP-seq and RNA-seq datasets. Visualizing this relationship in a four-quadrant plot revealed a predominantly positive correlation between m⁵C loss and transcriptional downregulation for the SHH pathway axis (Fig 2E). Notably, the downstream effector *Gli1* and the ligand *Ihh* fell into the lower-left quadrant (concurrent hypomethylation and downregulation), indicating a positive correlation between m⁵C loss and reduced transcript levels for these genes. Interestingly, a mechanistic divergence was observed for *Gli2*; while it exhibited the most dramatic loss of m⁵C modification (fold change = −7.40), its mRNA levels remained relatively stable.

The HH signaling pathway includes three ligands, SHH, IHH and Desert Hedgehog (DHH); two transmembrane protein receptors, PTCH and SMO; and the downstream transcription factors GLI1, GLI2, and GLI3 [8]. Previous studies have shown that IHH, a choroid-derived signal, can affect the retinal pigment epithelium (RPE), sclera, neural retina, and further retinal photoreceptors [26,27]. Based on their significant downregulation in our NMDA model and the established neuroprotective role of SHH signaling in the retina [28], we selected SHH and GLI1 for further validation. GLI2 was also included owing to its critical function in the HH signaling pathway and its significant m5C hypomodification, indicating its potential regulatory role at the epitranscriptomic level. Collectively, these data support our central hypothesis: NMDA-induced excitotoxicity impairs the SHH neuroprotective pathway by disrupting NSUN4-mediated m5C modification on its core transcripts. To test this, we proceeded with the following functional *in vitro* experiments.

### NSUN4 protects R28 cells from glutamate-induced excitotoxicity

**Fig 3:**
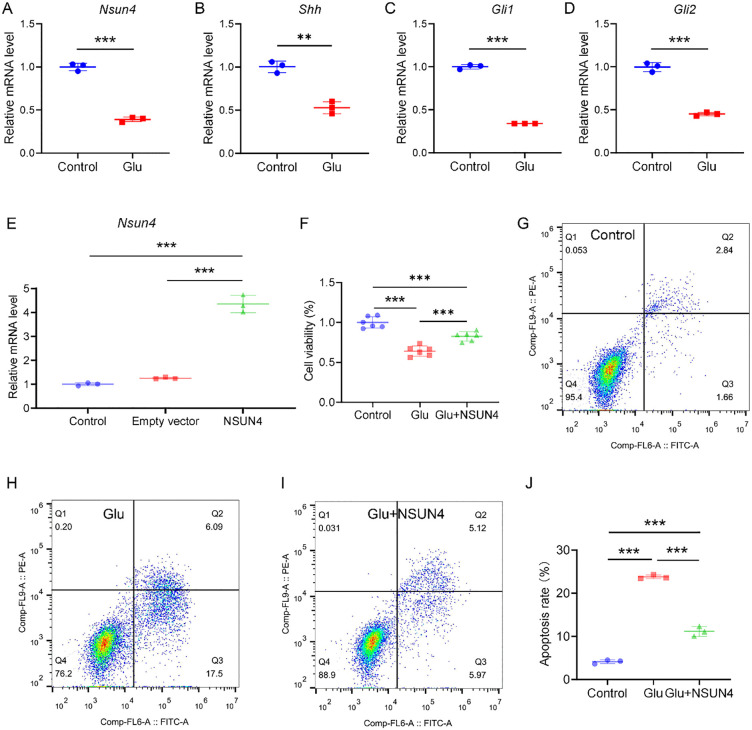
NSUN4 can protect R28 cells from glutamate excitotoxity. **A–D.** Quantitative real-time polymerase chain reaction (qRT‒PCR) analysis of the *Nsun4*, *Shh*, *Gli1*, and *Gli2* genes in the control and Glu groups. *β-Actin* was used as an internal control (**P < 0.01, ***P < 0.001 by two-tailed Student's *t*-tes*t*; Data are presented as mean ± SD; n = 3 biologically independent samples per group). **E.** qRT‒PCR analysis of the *Nsun4* genes in the control, empty vector, and NSUN4 groups. *β-Actin* was used as an internal control (***P < 0.001 by one-way ANOVA followed by Tukey's post hoc test; Results were Mean ± SD; n = 3 samples per group). **F.** CCK-8 assay to detect R28 cell viability after glutamate or NSUN4 exposure for 24 h (***P < 0.001 by one-way ANOVA followed by Tukey's post hoc test; Data are presented as mean ± SD; n = 6 technical replicates per group). **G–J.** NSUN4 reduced the apoptosis of R28 cells in the glutamate-induced model, as measured by Annexin V-FITC/PI (***P < 0.001 by one-way ANOVA followed by Tukey's post hoc test; Data are presented as mean ± SD; n = 3 biologically independent samples per group). Q1: living cells; Q2: early apoptotic cells; Q3: late apoptotic cells; Q4: necrotic cells. Apoptosis rate = number of cells in Q2 + Q3.

Since the mRNA level of *Nsun4*, an important m5C writer, was lower in the NMDA group than in the control group according to RNA-seq (Table 3), we further examined whether NSUN4 exerted regulatory effects on genes involved in the HH signaling pathway in glutamate-induced cell death in R28 cells. qRT‒PCR analysis revealed that glutamate treatment significantly decreased the mRNA level of *Nsun4* from 1.00 ± 0.04 in the control group to 0.39 ± 0.02 (*P* < 0.001; Fig 3A), a finding consistent with our *in vivo* RNA-seq results (Table 3). We also tested the mRNA levels of *Shh*, *Gli1*, and *Gli2* in the HH signaling pathway *in vitro* using qRT-PCR. As shown in Fig 3B–3D, the expression levels of these genes were significantly downregulated in R28 cells after treatment with glutamate (all *P* < 0.01). Together, these qRT‒PCR results confirm the downregulation of *Nsun4*, *Shh*, and *Gli1* in glutamate-injured R28 cells, validating our *in vivo* transcriptomic findings at the cellular level (Tables 3 and 5).

We then assessed NSUN4 levels in R28 cells with control, empty vector, or NSUN4 overexpression. qRT-PCR analysis revealed that NSUN4 overexpression markedly increased *Nsun4* mRNA levels to 4.35 ± 0.36. This value was approximately 4.3-fold and 3.5-fold higher than that in the control (1.00 ± 0.07) and empty vector (1.26 ± 0.04) groups, respectively (Fig 3E). Having confirmed successful NSUN4 overexpression in R28 cells, we next explored its protective role against apoptosis. Quantitatively, glutamate decreased viability to 64.2 ± 6.6% of the control level. NSUN4 overexpression significantly attenuated this cytotoxicity, restoring viability to 82.7 ± 5.7% (*P* < 0.001; Fig 3F), which corresponds to a recovery of approximately 51.6% of the glutamate‑induced loss. These results demonstrate that NSUN4 protects against glutamate-induced cytotoxicity.

Furthermore, Annexin V-FITC/PI double staining by flow cytometry was used to evaluate the effect of NSUN4 on cell mortality. Glutamate treatment significantly increased the total apoptosis rate to 23.80% ± 0.45% from a control level of 4.14% ± 0.45%. Notably, NSUN4 overexpression significantly attenuated this effect, reducing the apoptosis rate to 11.17% ± 1.15% (*P* < 0.001; Fig 3G–3J). The distribution of cells in the early and late apoptotic quadrants (Fig 3G–3I) confirmed that NSUN4's protection begins at the early stages of apoptosis.

### NSUN4 prevents glutamate-induced mitochondrial dysfunction and Ca^2*+*^ overload in R28 cells

**Fig 4:**
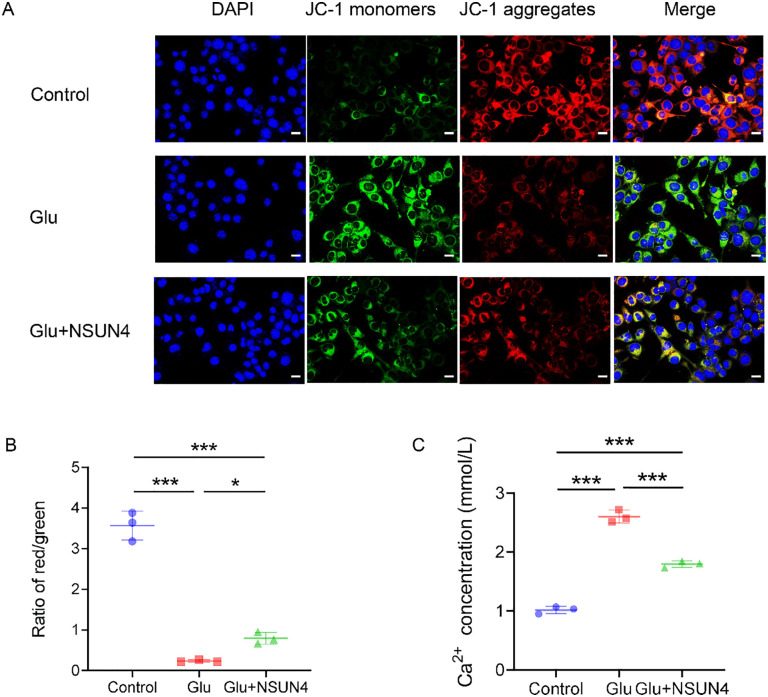
NSUN4 prevents mitochondrial dysfunction and Ca^2+^ overload in the Glu group. **A.** Representative fluorescence microscopy images of R28 cells stained with the JC-1 probe (scale bar = 20 μm). **B.** JC‑1 staining was used to assess mitochondrial membrane potential (MMP), represented as the ratio of red (aggregated JC‑1, high MMP) to green (monomeric JC‑1, low MMP) fluorescence intensity. *P < 0.05, ***P < 0.001 by one-way ANOVA followed by Tukey's post hoc test; Data are presented as mean ± SD; n = 3 biologically independent samples per group. **C.** Intracellular Ca^2+^ levels were evaluated and analyzed with a calcium colorimetric assay kit (***P < 0.001 by one-way ANOVA followed by Tukey's post hoc test; Data are presented as mean ± SD; Data are from three biologically independent samples per group). Glu, glutamate; NSUN4, NOP2/Sun RNA methyltransferase family member 4. Magnification: 400 × .

A decreased mitochondrial potential is an indicator of the early stage of cell apoptosis [29], and the MMP is believed to be an important parameter for assessing mitochondrial function [30]. We next evaluated the effect of NSUN4 on mitochondrial function by detecting the MMP in glutamate-treated R28 cells. According to the instructions of the JC-1 Assay Kit, the red and green fluorescence of R28 cells labeled with a JC-1 probe after treatment was observed by a fluorescence microscope (Fig 4A). The red/green ratio was deemed the relative value of the MMP. As shown in Fig 4B, the MMP decreased significantly in the glutamate group (0.24 ± 0.03) compared with that in the control group (3.57 ± 0.36) (*P* < 0.001). However, NSUN4 treatment partially reversed this glutamate-induced decrease, restoring the MMP to 0.79 ± 0.14 (*P* < 0.05) (Fig 4B). Apparently, NSUN4 has protective effects on mitochondria.

Glutamate-induced excitotoxicity is known to cause Ca² ⁺ overload and mitochondrial dysfunction, key drivers of neuronal death [31]. To determine if NSUN4 counteracts this pathway, we assessed its impact on intracellular Ca² ⁺ homeostasis. Glutamate exposure triggered a pronounced increase in intracellular Ca² ⁺ concentration (2.60 ± 0.11) relative to the control group (1.02 ± 0.06). Importantly, NSUN4 overexpression mitigated this glutamate-induced calcium dysregulation, maintaining a significantly lower concentration of 1.80 ± 0.06 (*P* < 0.001; Fig 4C). This ability to preserve Ca² ⁺ homeostasis underscores a key mechanism through which NSUN4 protects R28 cells. Overall, the ability of NSUN4 to preserve Ca² ⁺ homeostasis and mitigate mitochondrial dysfunction is closely associated with its protective effect against glutamate-induced excitotoxicity and apoptosis in R28 cells.

### NSUN4 increased the global level of m5C mRNA methylation in R28 cells induced by glutamate excitotoxicity

**Fig 5:**
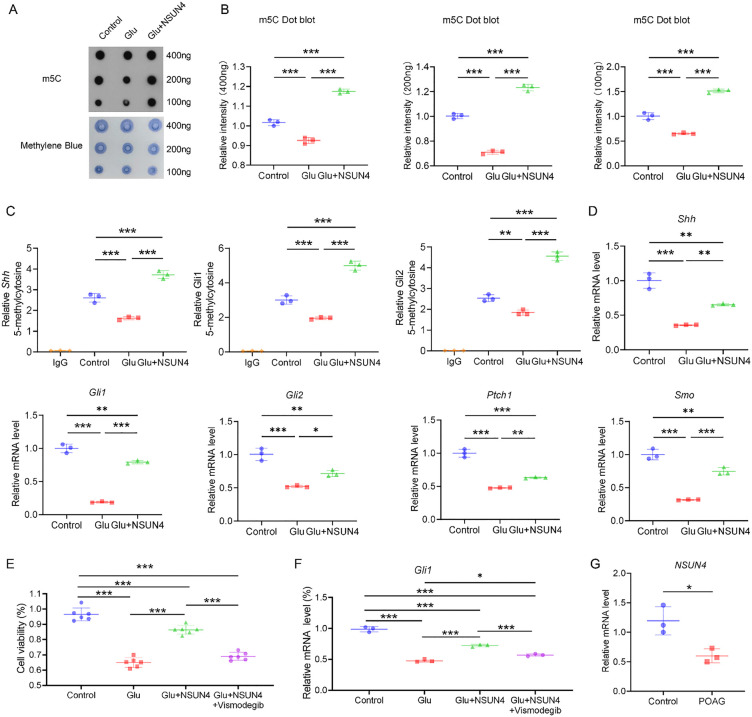
NSUN4 induced SHH signaling pathway methylation in glaucoma. **A.** The global m5C level of RNA extracted from R28 cells was measured via m5C dot blot assays. Equal amounts of RNA (400 ng, 200 ng and 100 ng) were loaded. The intensity of dot immunoblotting (top) was used to detect m5C modification, while methylene blue staining (bottom) represented the level of input RNA. **B.** The relative intensities of different amounts of RNA were analyzed (***P < 0.001 by one-way ANOVA followed by Tukey's post hoc test; Data are presented as mean ± SD; n = 3 biological replicates per group). **C.** The relative levels of m5C in *Shh*, *Gli1*, and *Gli2* mRNAs overexpressing NSUN4 were determined by MeRIP-qPCR using anti-m5C and anti-IgG antibodies. The IgG group was used as a negative control to prevent nonspecific binding. Statistical analysis (one-way ANOVA followed by Tukey’s post hoc test) was performed for comparisons among the Control, Glu, and Glu+NSUN4 groups (**P < 0.01, ***P < 0.001; Data are presented as mean ± SD; n = 3 biologically independent samples per group). **D.** Expression levels of the *Shh*, *Gli1*, *Gli2*, *Ptch1* and *Smo* genes in R28 cells after 10 mM glutamate treatment for 24 h with or without the addition of NSUN4 (*P < 0.05, **P < 0.01, ***P < 0.001 by one-way ANOVA followed by Tukey's post hoc test; Data are presented as mean ± SD; n = 3 biological replicates per group). **E.** NSUN4 overexpression rescued glutamate-induced damage, an effect reversed by the Hedgehog pathway inhibitor Vismodegib. CCK‑8 assay of cell viability after 24 h treatments. Data are mean ± SD (n = 6). Statistical significance was determined by one‑way ANOVA followed by Tukey's post‑hoc test: ***P < 0.001. **F.** NSUN4 activates the Hedgehog-GLI signaling axis under excitotoxic conditions. qRT‒PCR analysis of *Gli1* mRNA expression in the indicated treatment groups. *β-Actin* was used as an internal control. Data are presented as mean ± SD (n = 3). Statistical significance was determined by one-way ANOVA followed by Tukey’s post hoc test. *P < 0.05, **P < 0.01, ***P < 0.001 by one-way ANOVA followed by Tukey's post hoc test. **G.** Relative *NSUN4* mRNA expression levels in aqueous humor samples from patients with cataracts (Control) and patients with primary open-angle glaucoma (POAG), as determined by qRT-PCR. Data are presented as mean ± SD (n = 3 per group). Statistical significance was determined by an unpaired *t*-test (*P* < 0.05).

To investigate the role of RNA m⁵C modification in excitotoxic retinal ganglion cell death, we first assessed its global levels in a glutamate-induced R28 cell model using an m⁵C dot blot assay with serial RNA loads (400, 200, and 100 ng). Across all three loading concentrations, glutamate consistently reduced global mRNA m⁵C levels, while NSUN4 overexpression effectively rescued this reduction. Quantitative analysis of the 400 ng RNA load specifically showed that glutamate significantly reduced the global mRNA m⁵C level to 0.93 ± 0.02 compared to the control level of 1.02 ± 0.02 (*P* < 0.001). Crucially, NSUN4 overexpression not only reversed this reduction but elevated the m⁵C level to 1.18 ± 0.01 (*P* < 0.001; Fig 5A–5B). Having established that NSUN4 upregulates RNA m5C modification under excitotoxicity, we next asked whether it confers protection by specifically modulating the m5C levels of key Hedgehog pathway transcripts, namely *Shh*, *Gli1*, and *Gli2*.

### NSUN4 mitigates retinal neuronal excitotoxicity via m⁵C‑dependent activation of the Hedgehog-GLI signaling pathway

To substantiate the effect of NSUN4 on the m5C level of these genes, we first conducted MeRIP-qPCR using an anti-m5C antibody in R28 cells. MeRIP-qPCR indicated the *Shh*, *Gli1*, and *Gli2* mRNAs enrichment precipitated by m⁵C antibody. Glutamate exposure significantly reduced m⁵C enrichment on *Shh* (0.62-fold), *Gli1* (0.65-fold), and *Gli2* (0.73-fold) relative to control (all *P* < 0.001). However, NSUN4 overexpression robustly increased the m⁵C enrichment of *Shh*, *Gli1*, and *Gli2* mRNAs by approximately 2.3-fold compared to the glutamate-treated group (all *P* < 0.05) (Fig 5C). These results suggest that the protective effects of NSUN4 are associated with, and may involve, the enhancement of m⁵C modification on *Shh*, *Gli1*, and *Gli2* mRNAs.

We next asked if these m⁵C changes correlate with transcriptional regulation. qRT‒PCR confirmed that glutamate-induced excitotoxicity significantly suppressed the transcription of key Hedgehog pathway genes, reducing Shh, Gli1, and Gli2 mRNA levels to 36%, 19%, and 52% of control levels, respectively. NSUN4 overexpression significantly attenuated this transcriptional suppression, restoring expression to 65%, 79%, and 72% of control (*P* < 0.05; Fig 5D). We next examined the expression of the key Hedgehog signaling components Ptch1 (receptor) and Smo (signal transducer). Glutamate suppressed the expression of both Ptch1 and Smo. NSUN4 overexpression significantly counteracted the glutamate-induced suppression of Ptch1 and Smo, elevating their expression from 0.48 ± 0.01 to 0.63 ± 0.01 and from 0.32 ± 0.01 to 0.75 ± 0.06, respectively (*P* < 0.01) (Fig 5D). These results support the notion that NSUN4-mediated neuroprotection is associated with the upregulation of Hedgehog signaling pathway components, which occurs alongside increased m⁵C modification of their transcripts.

### Inhibition of the Hedgehog pathway abolishes the neuroprotective effect of NSUN4 in R28 cells

To further confirm that the neuroprotective effects of NSUN4 are indeed mediated through the activation of the Hedgehog-GLI signaling pathway, we performed a rescue experiment using Vismodegib, a potent and specific inhibitor of the Hedgehog pathway that acts by binding to Smo [32]. CCK-8 assays demonstrated that the marked enhancement in cell viability conferred by NSUN4 overexpression in the glutamate-induced excitotoxicity model was significantly abolished by Vismodegib treatment (Fig 5E). Quantitatively, NSUN4 overexpression increased cell viability by approximately 36.6% (from 65.0% in the Glu group to 88.8% in the Glu+NSUN4 group; *P* < 0.0001), whereas the addition of Vismodegib significantly reversed this protection, reducing viability back to 69.3% (*P* < 0.0001 vs. Glu+NSUN4 group), a level comparable to the glutamate-only group (*P* = 0.1937). These results demonstrate that blocking Hedgehog signaling abolishes the protective effect of NSUN4, confirming that pathway activation is essential for NSUN4‑mediated neuroprotection.

Consistent with the cell viability results, qRT-PCR analysis was conducted to evaluate the expression of *Gli1*, a reliable readout of Hedgehog pathway activity. Quantification of *Gli1* mRNA levels across treatment groups revealed a pattern of statistically significant changes (Fig 5F). Compared to the Control group, excitotoxic injury (Glu group) caused a significant downregulation of *Gli1* expression to approximately 48% of the baseline level (*P* < 0.001). This suppression was markedly reversed by NSUN4 overexpression, with the Glu+NSUN4 group showing a significant recovery to about 72% of the control level, representing a substantial increase compared to the Glu group (*P* < 0.001). Crucially, the administration of Vismodegib effectively attenuated this NSUN4-mediated rescue. The *Gli1* mRNA level in the Glu+NSUN4 + Vismodegib group was significantly lower than in the Glu+NSUN4 group (*P* = 0.0006), reverting to an intermediate level of approximately 57%. Notably, this level remained significantly higher than that in the Glu group alone (*P* = 0.0132), confirming a partial but specific inhibition of the pathway. Collectively, these findings provide strong evidence that NSUN4 exerts its protective influence on R28 cells by specifically modulating and maintaining the functional integrity of the Hedgehog-GLI signaling axis.

### The expression of Nsun4 mRNA was significantly downregulated in the aqueous humor of patients with primary open-angle glaucoma (POAG)

To evaluate the clinical relevance of this study, AH samples were collected from patients with cataracts (serving as controls) and patients with POAG. The demographic characteristics of the enrolled patients are summarized in Table 6. Demographic and baseline clinical characteristics were comparable between the POAG and control groups. Statistical analysis revealed no significant differences in age (*t*(4) = 2.292, *P* = 0.0836), gender distribution (Fisher's exact test, *P* > 0.999), or intraocular pressure (IOP; POAG: 19.00 ± 1.00 mmHg vs. Control: 19.00 ± 2.00 mmHg, *t*(4) = 0.000, *P* > 0.999). These resul*t*s indicate that the groups were well-matched, and these factors were unlikely to be confounding variables in this study. qRT-PCR results revealed a significant downregulation of *NSUN4* expression in the POAG group compared to the cataract control group (POAG: 0.60 ± 0.12 vs. Control: 1.19 ± 0.24; *P* < 0.05), representing an approximately 50% decrease (Fig 5G). These results suggest that the downregulation of *NSUN4* is specifically associated with the POAG pathological microenvironment, rather than a nonspecific response. Furthermore, the findings imply a potential compromise in the mitochondrial-related compensatory repair mechanisms in POAG, supporting the future development of *NSUN4* as a novel therapeutic target for neuroprotective strategies in glaucoma.

**Table 6:** Demographic and clinical characteristics of the study participants.

Characteristic	Control Group (n = 3)	POAG Group (n = 3)	Statistic	*P* value
Age (years, mean ± SD)	75.0 ± 3.6	69.3 ± 2.3	t(4) = 2.292	0.0836
Gender (Male/Female)	1/2	1/2	/	> 0.999
IOP (mmHg, mean ± SD)	19 ± 2.0	19 ± 1.0	t(4) = 0.000	> 0.999

Note: Data are mean ± SD (n = 3 per group). *P* values were derived from unpaired t-tests (Age, IOP) or Fisher’s exact test (Gender).

## Discussion

Glaucoma, a leading cause of irreversible blindness worldwide, is a neurodegenerative disease characterized by the dysfunction and ultimate loss of RGCs [1,33]. Current therapeutic strategies, which primarily focus on lowering intraocular pressure and providing neuroprotection, remain inadequate to completely halt RGC loss and prevent irreversible visual impairment [34]. Within the retina, glutamate serves as a critical neurotransmitter for visual signal transmission among photoreceptors, bipolar cells, and RGCs [35]. Under pathological conditions, however, excessive glutamate or the administration of NMDA can overstimulate NMDA receptors. This disrupts intracellular calcium homeostasis, triggering oxidative stress, inflammatory responses, and ultimately RGC death, all of which constitute the process termed glutamate excitotoxicity [34,36]. Mitochondrial dysfunction is a key consequence of this excitotoxicity, which is implicated in a range of neurodegenerative conditions including glaucoma, diabetic retinopathy, and ischemia-reperfusion injury [35,37]. Given its central role, understanding the mechanisms of RGC excitotoxicity in the early stages of glaucoma is crucial for developing effective treatments. A key mechanism underlying this excitotoxicity is the overactivation of NMDA receptors, which has been established as a pivotal driver of neuronal cell death [38]. The NMDA receptor 1 (NR1) subunit, essential for forming a functional NMDA receptor, is expressed throughout the retina, with widespread distribution in RGCs, amacrine cells, as well as bipolar cells [39]. Notably, nearly every neuron in the rat retinal ganglion cell layer expresses the NR1 subunit, regardless of developmental stage [39]. This ubiquitous expression underlies the particular vulnerability of these neurons to excitotoxic insult. Consequently, NMDA receptor overactivation directly led to the death of retinal neurons, particularly RGCs [38]. Supporting this, cells in ganglion cell layer of rabbit eyes have been shown to exhibit high sensitivity to NMDA. Collectively, these findings establish NMDA-mediated excitotoxicity as a critical factor in RGC death, making it a relevant model for simulating RGC damage in glaucoma [24,40,41]. Our previous study has demonstrated that intravitreal NMDA injection adversely affects the retinal *in vivo*: structurally, it reduced the thickness of the RGC and inner nuclear layers, caused the loss of RGCs, and altered RGC morphology; functionally, it impaired electrophysiological responses [24]. In addition to these changes, we also observed cytoplasmic organelle vacuolization in RGCs following NMDA injection compared to PBS-injected controls [24,42]. These findings align with previous reports, which demonstrated that NMDA induces similar morphological changes in RGCs and retinal dysfunction in mice [22,34].

RNA modifications are critical epigenetic regulators of biological processes [43]. mRNA modifications, including N6-methyladenosine (m6A), m5C, N1-methyladenosine (m1A), N7-methylguanosine (m7G), pseudouridine and uridylation, form the epitranscriptome to regulate protein synthesis [44–47]. In recent years, epigenetic studies, including studies on histone modifications, DNA methylation, noncoding RNAs, and m6A methylation, have been widely reported on the neuroprotective effects and regeneration of glaucoma [2]. For example, bioinformatics analysis of the TM tissues of POAG patients revealed that m6A regulatory genes could serve as potential diagnostic biomarkers for POAG [48]. Guan *et al*. [49] reported that m6A levels in the AH of patients with pseudoexfoliation glaucoma were significantly greater than those in patients with age-related cataracts. Methyltransferase 3 (METTL3), a m^6^A-related enzyme methylase, has the potential to increase the success rate of glaucoma filtration surgery [50]. While the role of m6A is increasingly recognized, the involvement of m5C remains largely unexplored in glaucoma. We performed high-throughput m5C MeRIP-seq and RNA-seq analyses in retinas following NMDA-induced excitotoxicity. This approach allowed us to identify, for the first time, the potential epigenetic alterations in m5C modification coupled with transcriptomic changes in an NMDA-induced excitotoxicity model. Through conjoint MeRIP-seq and RNA-seq analysis, we pinpointed the significant downregulation of NSUN4, an m5C writer known for its roles in mitochondrial metabolism [51]. Given its mitochondrial association, we hypothesized that NSUN4 promotes cell survival by mitigating excitotoxic mitochondrial damage.

**Fig 6:**
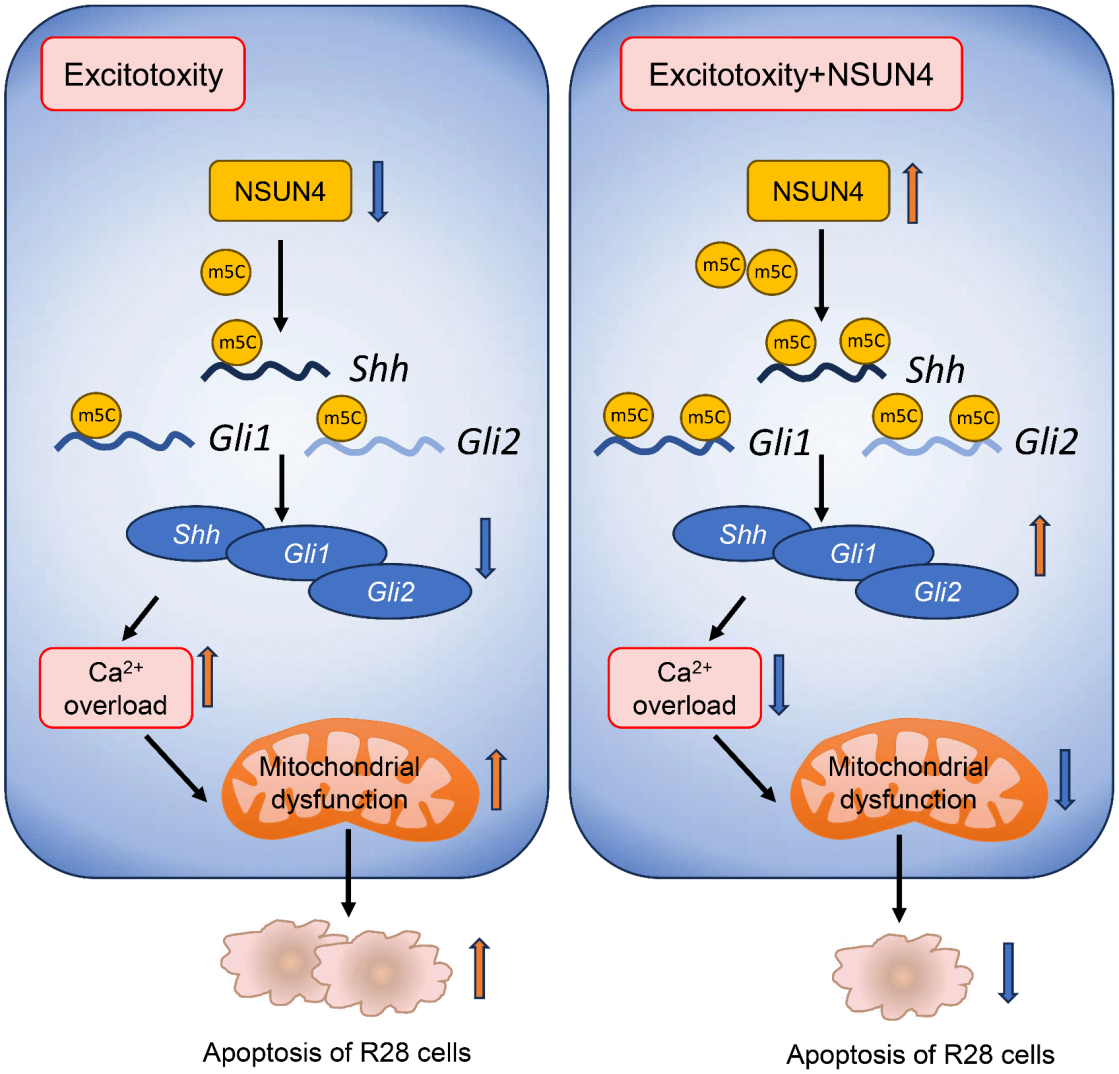
NSUN4 alleviates glutamate-induced injury in R28 cells via the m⁵C-SHH signaling axis.

To investigate this, we utilized the R28 cell line. Although an immortalized precursor line, R28 cells express the RGC marker 2G12 and possess functional NMDA receptor subunits, making them a validated model for mechanistic studies of excitotoxicity [52,53]. Due to these characteristics, R28 cells serve as a widely accepted and valuable tool for the mechanistic dissection of intracellular molecular processes, such as the m5C methylation machinery [21,22,54]. Their utility is further underscored by recent studies demonstrating that R28-derived biological components, such as extracellular vesicles, exert significant neuroprotective efficacy in glaucoma models [55]. These findings support the relevance of the R28 model for identifying therapeutic targets prior to validation in complex *in vivo* systems. We found that glutamate treatment causes cytosolic Ca² ⁺ overload, disruption of the MMP (an early apoptotic marker), and increased apoptosis—a phenomenon consistent with mitochondrial dysfunction in glaucomatous neurodegeneration [56]. Notably, the mitochondrial-related gene *NSUN4* was significantly downregulated in glutamate-treated cells [57]. Overexpression of NSUN4 effectively reversed these detrimental effects, alleviating excitotoxicity as evidenced by reduced Ca² ⁺ overload, improved mitochondrial function, and decreased apoptosis (Fig 6). These results demonstrate that NSUN4 overexpression mitigates glutamate-induced excitotoxicity, an effect closely associated with the preservation of mitochondrial function. Considering that previous research has ruled out the involvement of death receptor pathways, including tumor necrosis factor, c-Jun N-terminal kinases activation, or endoplasmic reticulum stress in NMDA-induced RGC death [58], our data collectively point to the preservation of mitochondrial integrity as a central mechanism underlying NSUN4-mediated neuroprotection. This is consistent with the established link between mitochondrial dysfunction and calcium-mediated RGC loss [59,60].

Given the functional importance of NSUN4 in neuroprotection and its bioinformatic association with mitochondria, we hypothesized that it might regulate specific survival signaling pathways. To identify such pathways, we performed an integrated analysis of transcriptomic and epitranscriptomic data. This analysis revealed a compelling link to the Hedgehog (HH) signaling pathway. MeRIP-seq revealed that genes with differential m5C hypomodification, including *Ihh*, *Gli1* and *Gli2*, were enriched in the HH signaling pathway. Consistent with this, RNA-seq showed downregulation of *Ihh*, *Shh* and *Gli1* in the NMDA-induced excitotoxicity model. Notably, we observed an upregulation of *Gli2* transcripts in the whole-retina RNA-seq data, which contrasts with the downregulation trend seen in the purified R28 cell model. This discrepancy suggests that the expression regulation of *Gli2* may be significantly influenced by additional, cell type-specific factors within the complex *in vivo* microenvironment. While the m5C modification of *Ihh* was more significantly altered than that of *Shh* in the whole retina, we chose to focus on SHH for further functional validation. This decision was based on the established and direct role of SHH, the best-studied HH ligand, in promoting RGC survival and protection against damage [61–63], whereas IHH's functions are more associated with the RPE and choroidal development [26,27]. Given that our MeRIP-seq data were generated from bulk retinal tissue, the profound change in *Ihh* m5C may originate from non-RGC components. Therefore, we prioritized SHH to specifically investigate its potential role in RGC excitotoxicity.

Previous studies have established the critical role of SHH in mitigating excitotoxicity, ischemic injury, and oxidative stress. For instance, the neuronal nitric oxide synthase-SRY-Box Transcription Factor 2-SHH axis can protect neurons against early excitotoxicity in cerebral ischemic stroke pathogenesis [64]. Additionally, SHH was found to promote neurite outgrowth in cortical neurons under H₂O₂-induced oxidative stress by counteracting ROS and mitochondrial dysfunction [9]. Having established its broad neuroprotective functions, we focus on its relevance to the retina and glaucoma. SHH signaling has been shown to decrease RGC loss in both diabetic and chronic ocular hypertension models, highlighting its neuroprotective potential in the retina [13,65]. Furthermore, genetic evidence from whole-exome sequencing and Sanger sequencing identifies SHH as a potential risk factor in glaucoma patients [66]. Beyond RGCs, the SHH pathway also regulates the function of trabecular meshwork cells, a key tissue in glaucoma pathogenesis, by mediating GLI1, suggesting GLI1 as a potential therapeutic target [14].

In line with these findings, our present study confirms that glutamate disruption in R28 cells significantly downregulates the expression of *Shh*, and *Gli1*. Given the cell-type-specific expression pattern noted above, the downregulation of *Gli2* in this neuronal-like cell line further supports its context-dependent regulation. Furthermore, we observed downregulation of the key receptors Ptch and Smo, which form the critical membrane complex that initiates SHH signal transduction [8]. This broad suppression of pathway components solidifies the involvement of the SHH pathway in our excitotoxicity model. Having established the protective role of NSUN4, we sought to determine whether it is associated with increased m5C modification of *Shh*, *Gli1*, and *Gli2* transcripts. Dot blot analysis confirmed that global mRNA m5C levels were elevated upon NSUN4 overexpression. To probe the specific genes involved, we performed MeRIP-qPCR, which revealed significant enrichment of *Shh*, *Gli1*, and *Gli2* mRNAs with the m5C antibody, confirming that these transcripts are m5C-modified. As predicted, the m5C modification levels on these transcripts were significantly reduced in the NMDA-treated group, and this reduction was rescued by NSUN4 overexpression. Collectively, these results suggest that NSUN4 overexpression elevates global m5C levels and, specifically, the m5C modification levels on *Shh*, *Gli1*, and *Gli2* mRNAs in R28 cells (Fig 5C). Critically, the functional necessity of this regulatory axis was confirmed by pharmacological inhibition as the neuroprotective effect of NSUN4 was substantially reversed following blockade of the SHH pathway with Vismodegib (Fig 5M). Based on these converging lines of evidence, we propose that NSUN4 confers neuroprotection, at least in part, by enhancing m⁵C modification on SHH pathway transcripts, thereby sustaining pathway activity and promoting cell survival (Fig 6). While this study establishes the NSUN4‑m⁵C‑SHH axis as a causally linked and necessary pathway for the observed protection, it is important to consider that NSUN4‑mediated neuroprotection may involve additional molecular mechanisms. As an m⁵C writer, NSUN4 could potentially influence other survival pathways critical for retinal ganglion cells, such as those regulating mitochondrial biogenesis or translational control of pro‑survival factors [67,68]. Thus, the full protective phenotype may arise from the coordinated action of multiple epitranscriptomic programs, with the SHH pathway representing a central, identified effector.

Importantly, the pathophysiological relevance of NSUN4 downregulation extends beyond our cellular model. Our clinical data from POAG patients demonstrated a significant reduction of NSUN4 in the aqueous humor, indicating a potential association between NSUN4 downregulation and the glaucomatous condition. We acknowledge that aqueous humor provides an indirect though relevant readout; future studies using human retinal tissue or single cell analysis will be essential to confirm the specific downregulation of NSUN4 in RGCs and to establish a more direct tissue level link. Nevertheless, this clinical observation, together with our mechanistic data, positions NSUN4 as a promising candidate worthy of further investigation for neuroprotective intervention in glaucoma.

This study has several limitations. Firstly, our mechanistic investigations were primarily conducted in the R28 cell line. Although this provides a stable model for dissecting the NSUN4-SHH axis under excitotoxicity, immortalized cells may not fully mirror the physiological complexity of mature RGCs. Subsequent studies in primary RGC cultures and chronic ocular hypertension models will be instrumental in confirming the long-term pathophysiological relevance of these findings. Secondly, our current omics approach utilized bulk retinal tissues, which provides a comprehensive molecular overview but lacks the resolution to distinguish cell-type-specific responses. Since RGCs represent only a small fraction of the total retinal cell population, their specific epitranscriptomic signatures may be partially diluted by signals from other cell types. The integration of single-cell m5C profiling and spatial transcriptomics in future studies will be crucial to precisely map the spatial and cell-specific regulatory landscape of NSUN4 in the retina. Finally, the necessity of NSUN4 itself requires confirmation through knockdown approaches, and the spatial regulation of this axis within retinal tissue remains to be visualized. Furthermore, elucidating the specific mechanisms by which m⁵C modification influences the translation or stability of key effectors will provide a more granular understanding of the epitranscriptomic circuitry underlying retinal neuroprotection.

## Conclusion

This study presents an initial profile of RNA m5C modifications in NMDA-induced retinal injury, offering a resource for future investigation. Our findings indicate that NSUN4, an m5C methyltransferase, may protect R28 cells from excitotoxicity by reducing Ca² ⁺ overload, mitochondrial dysfunction, and apoptosis. Mechanistically, we establish that NSUN4 exerts this protection by specifically activating the Hedgehog-GLI signaling pathway. This is evidenced by the NSUN4-dependent enhancement of m⁵C methylation on key pathway transcripts (*Shh* and *Gli1*) and the consequential upregulation of their expression. Crucially, the neuroprotective effect of NSUN4 was abolished upon pharmacological inhibition of the pathway with Vismodegib, providing direct functional validation of this causal linkage. The clinical relevance of this axis is underscored by the significant downregulation of *NSUN4* in the aqueous humor of patients with primary open-angle glaucoma. Collectively, our findings define NSUN4-mediated m⁵C modification as an upstream regulatory mechanism that sustains Hedgehog-GLI signaling, thereby nominating it as a promising target for developing novel neuroprotective strategies against glaucomatous neurodegeneration.

**Key message**

NSUN4 acts as a critical m^5^C methyltransferase that mitigates glutamate-induced excitotoxicity in retinal cells. It functions by enhancing m5C RNA methylation on key transcripts of the SHH signaling pathway, including *Shh*, *Gli1*, and *Gli2*.

Overexpression of NSUN4 effectively reverses mitochondrial dysfunction, attenuates intracellular Ca^2+^ overload, and inhibits apoptosis in glutamate-treated R28 cells.

*NSUN4* levels are significantly reduced in the aqueous humor of POAG patients, highlighting the clinical relevance of NSUN4-mediated m⁵C regulation in glaucomatous neurodegeneration.

The NSUN4-m5C-SHH regulatory axis represents a novel epigenetic mechanism for maintaining retinal neuronal homeostasis, identifying NSUN4 as a potential therapeutic target for neuroprotective intervention in glaucoma.

## Supporting information

S1 Table Cloning primer sequences of Nsun4 genes. (PDF)

S2 Table The list of m5C regulator genes in the MeRIP-seq. (PDF)
