# Supplementary material for: NSUN4-mediated m5C RNA methylation protects retinal cells against excitotoxic injury via the SHH signaling pathway
Source: PLoS One. 2026 Apr 15;21(4):e0347414. doi: 10.1371/journal.pone.0347414 (PMC13082710; doi:10.1371/journal.pone.0347414)
Supplement: S1 Table — (PDF) [file pone.0347414.s001.pdf]

**S1 Table. Cloning primer sequences of Nsun4 genes.**

| Genes | | Sequences（5’-3’） |
|---|---|---|
| *Nsun4* | Forward | TTAAACTTAAGCTTGGTACCATGGCTGCGCCCGTATTAAG |
| | Reverse | CACACTGGACTAGTGGATCCCTATGGCACCCTGTGCAATT |
