# Supplementary material for: NSUN4-mediated m5C RNA methylation protects retinal cells against excitotoxic injury via the SHH signaling pathway
Source: PLoS One. 2026 Apr 15;21(4):e0347414. doi: 10.1371/journal.pone.0347414 (PMC13082710; doi:10.1371/journal.pone.0347414)
Supplement: S2 Table — (PDF) [file pone.0347414.s002.pdf]

S2 Table. The list of m5C regulator genes in the MeRIP-seq.

gene	gene_id	biotype	strand	locus	NMDA.Input_FPKM	Normal.Input_FPKM	Fold change	log2(fold change)	p_value	FDR	Regulation	GeneID	Synonyms	dbXrefs	chromosome	map_location	description	GO-CC	GO-MF	GO-BP
Nsun4	ENSRNOG00000012020	protein_coding	-	chr5:138153150-138173265	0.0766	0.24349	-3.179	-1.668	1	1	down	298426	RGD1559652	RGD:1559652|Ensembl:ENSRNOG00000046192	5	5q36	NOP2/Sun RNA methyltransferase family member 4	GO:0005739(mitochondrion)//GO:0005575(cellular_component)//GO:0005762(mitochondrial large ribosomal subunit)	GO:0003674(molecular_function)//GO:0008168(methyltransferase activity)//GO:0070181(small ribosomal subunit rRNA binding)	GO:0070131(positive regulation of mitochondrial translation)//GO:0031167(rRNA methylation)//GO:0042256(mature ribosome assembly)//GO:0008150(biological_process)
Nsun5	ENSRNOG00000001450	protein_coding	-	chr12:26339242-26344203	4.09183	5.97155	-1.459	-0.545	0.46	1	down	288595	RGD1309268	RGD:1309268|Ensembl:ENSRNOG00000001450	12	12q12	NOP2/Sun RNA methyltransferase	GO:0005730(nucleolus)//GO:0005634(nucleus)	GO:0044822(poly(A) RNA binding)//GO:0008168(methyltransferase activity)	GO:0032259(methylation)

																	family member 5			
Tet1	ENSRNOG00000000277	protein_coding	+	chr20:29197549-29267359	4.74115	6.72202	-1.4118	-0.504	0.3	1	down	309902	Cxxc6	RGD:1310993|Ensembl:ENSRNOG00000000277	20	20p11	tet methylcytosine dioxygenase 1	GO:0005634(nucleus)//GO:0005575(cellular_component)	GO:0003674(molecular_function)//GO:0008270(zinc ion binding)//GO:0070579(methylcytosine dioxygenase activity)//GO:0005506(iron ion binding)//GO:0043566(structure-specific DNA binding)//GO:0003677(DNA binding)	GO:0055114(oxidation-reduction process)//GO:0008284(positive regulation of cell proliferation)//GO:0080111(DNA demethylation)//GO:0044030(regulation of DNA methylation)//GO:2000653(regulation of genetic imprinting)//GO:0031062(positive regulation of histone methylation)//GO:0006493(protein O-linked glycosylation)//GO:0045944(positive regulation of transcription from RNA polymerase II promoter)//GO:0008150(biological_process)//GO:0090310(negative regulation of methylation-

																				dependent chromatin silencing)//GO:0019827(stem cell population maintenance)//GO:0001826(inner cell mass cell differentiation)
Nsun2	ENSRNOG00000017254	protein_coding	-	chr1:37692671-37716925	6.56682	8.16251	-1.2443	-0.3144	0.57	1	down	361191	RGD1311954	RGD:1311954|Ensembl:ENSRNOG00000017254	1	-	NOP2/Sun RNA methyltransferase family, member 2	GO:0005730(nucleolus)//GO:0005634(nucleus)//GO:0005737(cytoplasm)//GO:0033391(chromatoid body)	GO:0044822(poly(A) RNA binding)//GO:0016428(tRNA (cytosine-5-)-methyltransferase activity)//GO:0000049(tRNA binding)	GO:0030488(tRNA methylation)//GO:0007286(spermatid development)//GO:0033313(meiotic cell cycle checkpoint)
Nsun4	ENSRNOG00000046192	protein_coding	-	chr5:138670429-138689188	0.36861	0.3961	-1.075	-0.104	1	1	down	298426	RGD1559652	RGD:1559652|Ensembl:ENSRNOG00000046192	5	5q36	NOP2/Sun RNA methyltransferase family member 4	GO:0005739(mitochondrion)//GO:0005575(cellular_component)//GO:0005762(mitochondrial large ribosomal subunit)	GO:0003674(molecular_function)//GO:0008168(methyltransferase activity)//GO:0070181(small ribosomal subunit rRNA binding)	GO:0070131(positive regulation of mitochondrial translation)//GO:0031167(rRNA methylation)//GO:0042256(mature ribosome assembly)//GO:0008150(biological_process)

Alyref	ENSRNOG00000036687	protein_coding	-	chr10:109363928-109367692	24.7919	25.0005	-1.008	-0.012	0.98	1	down	690585	Thoc4	RGD:1594679|Ensembl:ENSRNOG00000036687	10	10q32.3	Aly/REF export factor	GO:0000346(transcription export complex)//GO:0070062(extracellular exosome)//GO:0035145(exon-exon junction complex)//GO:0071013(catalytic step 2 spliceosome)//GO:0016020(membrane)//GO:0005575(cellular_component)//GO:0000784(nuclear chromosome, telomeric region)//GO:0043231(intracellular membrane-bounded organelle)//GO:0005654(nucleoplasm)//GO:0005634(nucleus)	GO:0003674(molecular_function)//GO:0003723(RNA binding)//GO:0003676(nucleic acid binding)//GO:0000166(nucleotide binding)//GO:0044822(poly(A) RNA binding)//GO:0003697(single-stranded DNA binding)	GO:0006406(mRNA export from nucleus)//GO:0000018(regulation of DNA recombination)//GO:0046784(viral mRNA export from host cell nucleus)//GO:0032786(positive regulation of DNA-templated transcription, elongation)//GO:0031297(replication fork processing)//GO:0001649(osteoblast differentiation)//GO:0008150(biological_process)
Tet3	ENSRNOG000	protein_co	-	chr4:17965075	93.6717	93.1953	1.00	0.0074	0.99	1	up	680	-	RGD:1590584|Ensembl:EN	4	4q34	tet methylcytosin	GO:0005575(cellular_component)//GO:0005634(nucleus)//G	GO:0003674(molecular_function)//GO:0008270	GO:0055114(oxidation-reduction process)//GO:0080111(D

	00011387	ding		8-179747431			51					576		SRNOG00000011387			e dioxygenase 3	O:0001939(female pronucleus)//GO:0001940(male pronucleus)//GO:0005737(cytoplasm)	(zinc ion binding)//GO:0070579(methylcytosine dioxygenase activity)//GO:0003677(DNA binding)	NA demethylation)//GO:0080182(histone H3-K4 trimethylation)//GO:0006493(protein O-linked glycosylation)//GO:0045944(positive regulation of transcription from RNA polymerase II promoter)//GO:0008150(biological_process)//GO:0044727(DNA demethylation of male pronucleus)
Tet2	ENSRNOG00000023579	protein_coding	-	chr2:257255361-257279069	25.3291	24.9502	1.01552	0.0217	0.95	1	up	310859	RGD1311625	RGD:1311625|Ensembl:ENSRNOG00000023579	2	2q43	tet methylcytosine dioxygenase 2	GO:0005575(cellular_component)	GO:0003674(molecular_function)//GO:0008270(zinc ion binding)//GO:0070579(methylcytosine dioxygenase activity)//GO:0008198(ferrous iron binding)	GO:0055114(oxidation-reduction process)//GO:0020027(hemoglobin metabolic process)//GO:0061484(hematopoietic stem cell homeostasis)//GO:0080111(DNA demethylation)//GO:0019857(5-methylcytosine metabolic process)//GO:0080182(hi

stone H3-K4 trimethylation)//GO:0006211(5-methylcytosine catabolic process)//GO:0048872(homeostasis of number of cells)//GO:0009791(post-embryonic development)//GO:0030099(myeloid cell differentiation)//GO:0006493(protein O-linked glycosylation)//GO:0030097(hemopoiesis)//GO:0002318(myeloid progenitor cell differentiation)//GO:0014070(response to organic cyclic compound)//GO:0072576(liver morphogenesis)//GO:0001822(kidney development)//GO:0048536(spleen development)//GO:00198

																				58(cytosine metabolic process)//GO:0045944(positive regulation of transcription from RNA polymerase II promoter)
Ybx1	ENSRNOG00000023786	protein_coding	-	chr5:142136440-142152428	250.056	239.371	1.0446	0.063	0.87	1	up	500538	Byb1|Cbfa|Dbpb|Ef1a|Msy1|Nsep1|Yb1	RGD:61843|Ensembl:ENSRNOG00000023786	5	5q36	Y box binding protein 1	GO:0030529(intracellular ribonucleoprotein complex)//GO:0005576(extracellular region)//GO:0030425(dendrite)//GO:0048471(perinuclear region of cytoplasm)//GO:0071204(histone pre-mRNA 3'end processing complex)//GO:0005634(nucleus)//GO:1990124(messenger ribonucleoprotein complex)//GO:0005737(cytoplasm)//GO:0010494(cytoplasmic stress granule)	GO:0003677(DNA binding)//GO:0003723(RNA binding)//GO:0003682(chromatin binding)//GO:0003729(mRNA binding)//GO:0008134(transcription factor binding)//GO:0043565(sequence-specific DNA binding)//GO:0003697(single-stranded DNA binding)//GO:0005515(protein binding)//GO:0	GO:0008284(positive regulation of cell proliferation)//GO:0008380(RNA splicing)//GO:0043066(negative regulation of apoptotic process)//GO:0006351(transcription, DNA-templated)//GO:0001701(in utero embryonic development)//GO:0046627(negative regulation of insulin receptor signaling pathway)//GO:0051154(negative regulation of striated muscle cell differentiation)//GO:0045944(positive regulation of transcription from RNA polymerase II

																			002039(p53 binding)	promoter)//GO:0000122(negative regulation of transcription from RNA polymerase II promoter)//GO:0006397(mRNA processing)//GO:0051781(positive regulation of cell division)//GO:0070934(CRD-mediated mRNA stabilization)//GO:0006355(regulation of transcription, DNA-templated)
Ythdf2	ENSRNOG00000010892	protein_coding	-	chr5:154038416-154062397	29.2747	27.6275	1.05596	0.0835	0.85	1	up	313053	-	RGD:1311321|Ensembl:ENSRNOG00000010892	5	5q36	YTH N(6)-methyladenosine RNA binding protein 2	GO:0000932(cytoplasmic mRNA processing body)//GO:0005634(nucleus)//GO:0005737(cytoplasm)//GO:0005829(cytosol)	GO:0044822(poly(A) RNA binding)//GO:1990247(N6-methyladenosine-containing RNA binding)	GO:0043488(regulation of mRNA stability)//GO:1903679(positive regulation of cap-independent translational initiation)
Nsun	ENSRNO	protein	-	chr17:83	3.86111	2.94572	1.3	0.39	0.6	1	up	30	Nopd1	RGD:1309535|Ens	17	17q12.3	NOP2/Sun		GO:0003723(RNA	GO:0032259(methylation)

6	G00000018520	_coding		76447 6-83802110			108	04	2			71488		embl:ENSRNOG00000018520			RNA methyltransferase family member 6	binding)//GO:0008168(methyltransferase activity)	
Nsun7	ENSRNOG00000025494	protein_coding	-	chr14:43346770-43374401	0.75852	0.48697	1.55577	0.6394	1	1	up	305339	RGD1310137	RGD:1310137|Ensembl:ENSRNOG00000025494	14	14p11	NOP2/Sun RNA methyltransferase family member 7	GO:0008168(methyltransferase activity)	GO:0030382(sperm mitochondrion organization)//GO:0030317(sperm motility)//GO:0032259(methylation)
